# Single‐Cell‐Driven Tri‐Channel Encryption Meta‐Displays

**DOI:** 10.1002/advs.202203962

**Published:** 2022-10-26

**Authors:** Muhammad Qasim Mehmood, Junhwa Seong, Muhammad Ashar Naveed, Joohoon Kim, Muhammad Zubair, Kashif Riaz, Yehia Massoud, Junsuk Rho

**Affiliations:** ^1^ MicroNano Lab Electrical Engineering Department Information Technology University (ITU) of the Punjab Ferozepur Road Lahore 54600 Pakistan; ^2^ Department of Mechanical Engineering Pohang University of Science and Technology (POSTECH) Pohang 37673 Republic of Korea; ^3^ Innovative Technologies Laboratories (ITL) King Abdullah University of Science and Technology (KAUST) Thuwal 23955 Saudi Arabia; ^4^ Department of Chemical Engineering Pohang University of Science and Technology (POSTECH) Pohang 37673 Republic of Korea; ^5^ POSCO‐POSTECH‐RIST Convergence Research Center for Flat Optics and Metaphotonics Pohang 37673 Republic of Korea; ^6^ National Institute of Nanomaterials Technology (NINT) Pohang 37673 Republic of Korea

**Keywords:** high‐dense meta‐optics, meta‐displays, metahologram, phase‐amplitude modulation, tri‐functional metasurface

## Abstract

Multi‐functional metasurfaces have attracted great attention due to the significant possibilities to realize highly integrated and ultra‐compact meta‐devices. Merging nano‐printing and holographic information multiplexing is one of the effective ways to achieve multi‐functionality, and such a merger can increase the information encoding capacity. However, the current approaches rely on stacking layers and interleaving, where multiple resonators effectively combine different functionalities on the cost of efficiency, design complexity, and challenging fabrication. To address such challenges, a single meta‐nanoresonator‐based tri‐functional metasurface is proposed by combining the geometric phase‐based spin‐decoupling and Malus's law intensity modulation. The proposed strategy effectively improves information capacity owing to the orientation degeneracy of spin‐decoupling rather than layer stacking or super‐cell designs. To validate the proposed strategy, a metasurface demonstrating two helicity‐dependent holographic outputs is presented in far‐field, whereas a continuous nano‐printing image is in near‐field. It is also employed on CMOS‐compatible and cost‐effective hydrogen amorphous silicon providing transparent responses for the whole visible band. As a result, the proposed metasurface has high transmission efficiency in the visible regime and verifies the design strategy without adding extra complexities to conventional nano‐pillar geometry. Therefore, the proposed metasurface opens new avenues in multi‐functional meta‐devices design and has promising applications in anti‐counterfeiting, optical storage and displays.​

## Graphical abstract

A single meta‐atom‐based tri‐functional metasurface is designed and experimentally demonstrated by combining the geometric phase‐based spin‐decoupling and Malus's law intensity modulation. This method proposes an effective way toward information multiplexing by combining holography and nanoimprinting in a single metasurface. The proposed metasurface can pave the way for multi‐functional meta‐devices with high‐density information.

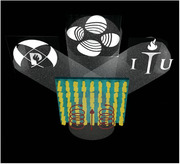

## 1 Introduction

Artificially engineered planar materials, often called metasurfaces, have proven their unprecedented ability to manipulate intrinsic properties of incident light such as amplitude, polarization, and phase at the subwavelength scale.^[^ ^1^, ^2^, ^3^, ^4^, ^5^, ^6^, ^7^, ^8^, ^9^, ^10^, ^11^, ^12^ ^]^ Nanoscale unit elements provide them with exceptional light steering capability and open new avenues in imaging technology where they are employed to record nano‐printing images and project holographic images with high resolution and efficiency.^[^ ^13^, ^14^, ^15^, ^16^, ^17^, ^18^, ^19^ ^]^ Recently, several design techniques have been proposed to independently manipulate the near and far‐field of incident light.^[^ ^20^, ^21^ ^]^ These near‐field holograms are referred to as Fresnel holograms, and their propagation distance is sub‐wavelength scale, whereas far‐field holograms are called Fourier‐type holograms, and their propagation distance is far greater compared to operating wavelength. Different design approaches like segmenting,^[^ ^22^ ^]^ layer stacking,^[^ ^23^ ^]^ interleaving,^[^ ^24^ ^]^ and phase‐mergence have been reported previously.^[^ ^25^ ^]^ Although the functionalities of all aforementioned techniques are improved from single to multiple functionalities, still most of them are actually single function performing devices since different operational zones are performing a single functionality. Resultantly, the light parameter (polarization, wavelength, etc.) that works well for one functionality is actually noise for the other, which lowers the device efficiency.

Merging the far‐field meta‐holography and near‐field nano‐printing on a single metasurface interface is an emerging technique to further increase the information encryption capacity.^[^ ^26^, ^27^ ^]^ The fundamental of this approach lies in intelligently engineering the amplitude and phase of incident waves simultaneously. For example, by stacking multi‐layers design on the front and rear face or using an interleaving design approach, the phase and amplitude of the incident wave can be manipulated for both far‐ and near‐field imaging displays.^[^ ^28^, ^29^, ^30^ ^]^ However, information encryption density is not increased in reality with these approaches. By exploiting the in‐between coupling of two nanoresonators, Bao et al. reported complex transmission coefficients adjustment technique to realize meta‐holography and nano‐printing.^[^ ^13^ ^]^ Similarly, Overvig et al. employed the phase delay between optimized varying dimensions nanoresonators to achieve a multi‐functional metasurface.^[^ ^31^ ^]^ However, the amplitude distribution of the transmitted field (which determines the nano‐printing pattern) limits the diffraction efficiency of the far‐field meta‐hologram, which is a major drawback of the above‐mentioned techniques. A unification of retardation and geometric phase and continuous amplitude tuning technique has been introduced to mitigate these efficiency issues. The proposed technique employed an array of varying dimensions nanoresonators with a fixed in‐between phase difference.^[^ ^26^, ^27^ ^]^ Although the proposed technique solves the efficiency problem, however, dispersion issues in broadband response and challenging fabrication process due to varying dimensions of nanoresonators are major hindrances of this technique. Therefore, there is a strong need to develop a simple design strategy that can effectively address all the design, fabrication, and efficiency issues.

**Figure 1:**
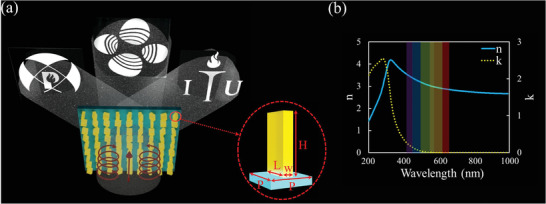
Schematics of the proposed single meta‐nanoresonator‐based trifunctional metasurface. a) When LHCP light is incident, it produces a holographic image of “ITU Logo”. When RHCP light is incident, it produces a holographic image of “POSTECH Logo”. However, when linearly horizontally polarized (x‐LP) is shined, and metasurface is analyzed through a *y*‐polarizer, it produces a holographic image of the “KAUST Logo”. All holographic outputs are displayed on a central symmetric plane; they are shown as off‐axis, just for ease of display here. Meta‐nanoresonator, which is a fundamental building block of the tri‐functional metasurface, is depicted in the inset. The optimal values of the physical parameters are *L* = 220 nm, *w* = 90 nm, *P* = 290 nm, and *H* = 400 nm. b) Measured “n” and “k” of the newly optimized material a‐Si:H are depicted.

Here, we propose a novel and simple design approach which combines the spin‐decoupling strategy with intensity modulation governed by Malus's law.^[^ ^32^, ^33^ ^]^ We employed a single anisotropic meta‐nanoresontor (fixed length, width, height, and period) made of a‐Si:H/SiO_2_ to demonstrate three‐channel information multiplexing. In order to achieve a transparent window for the whole visible domain, we further optimized the conventional hydrogenated amorphous silicon (a‐Si:H) with higher hydrogen content.^[^ ^34^ ^]^ Measured ellipsometry “*n*” and “*k*” of newly optimized a‐Si:H is depicted in Figure 1b. Unlike the previously reported phase‐mergence technique, we employed only the geometric phase of a single anisotropic nanoresonator to encode three independent pieces of information on the metasurface interface. All three encoded information can be decoded with different incident polarization scenarios and optical set‐ups, which acts as a decoding key. When left‐hand circularly polarized (LHCP) and right‐hand circularly polarized (RHCP) are incident proposed metasurface projects “ITU” and “POSTECH” logos in the far‐field plane, whereas when linearly polarized light is shined, and proper optical set‐up is installed, a gray‐scale hologram of “KAUST” logo is displayed in the near‐field (**Figure** **1**a). The proposed technique provides an efficient solution to multiplex multiple information on the metasurface interface without entangling design and fabrication complexities. This makes the proposed technique a potential contender for many multi‐fold and high‐end anti‐counterfeiting, information‐hiding, and high‐dense optical storage applications.

## 2 Experimental Section

### 2.1 Design Principle of Tri‐Functional Metasurface

**Figure 2:**
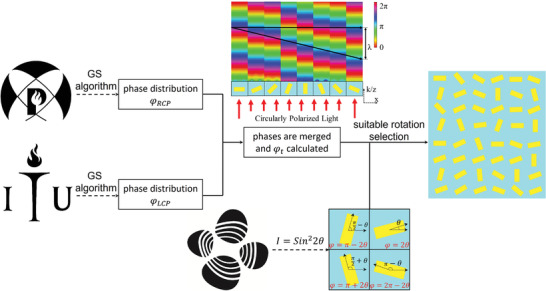
Detailed designing flow diagram of the tri‐functional metasurface. First two high‐resolution logos of “POSTECH” and “ITU” are selected, and their phase distributions ****φ****_**RCP**_ and ****φ****_**LCP**_ are calculated through the GS algorithm. These calculated phases are merged by processing through Equation (3). By carefully optimizing the anisotropic meta‐nanoresonator, a complete 0–2*π* phase is achieved by varying its in‐plane orientation. Meanwhile, we calculated an intensity profile for a targeted near‐field Fresnel image, and its orientations are discretized. By carefully selecting required orientations proposed tri‐functional metasurface is designed.

**Figure 3:**
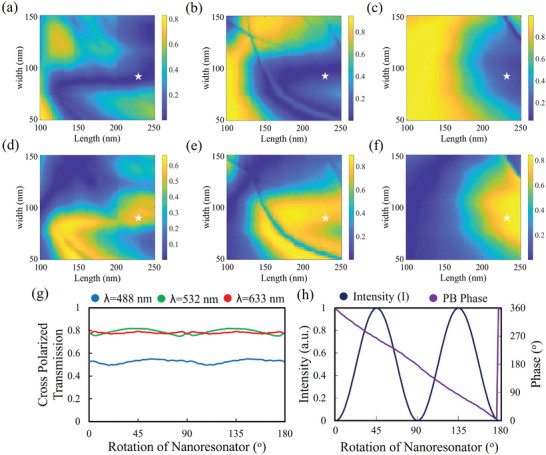
Optimization of a‐Si:H meta‐nanoresonator. Simulated co‐polarized and cross‐polarized transmission efficiencies are depicted for different widths (w) and lengths (L). a–c) depicts co‐polarized transmission efficiency at *λ* = 488 nm, *λ* = 532 nm, and *λ* = 633 nm, respectively. d–f) depicts cross‐polarized transmission efficiency at *λ* = 488 nm, *λ* = 532 nm, and *λ* = 633 nm, respectively. Selected meta‐nanoresonator (dimension is highlighted with white star) have minimum copolarized transmission and maximum cross‐polarized transmission at all operating wavelengths. To achieve complete 0–2*π* phase coverage selected meta‐nanoresonator is rotated (0–2*π*) about its axis. g) depicts cross‐polarization transmission, and h) depicts complete phase coverage along with continuous near‐field amplitude tuning of the selected meta‐nanoresonator.

In order to achieve a tri‐functional metasurface, we combined the geometric phase manipulation and amplitude modulation governed by Malus's law (**Figure** **2**). Each nanoresonator behaves as a half wave‐plate and rotates the polarization of linearly polarized (LP) incident light. Therefore, it can manipulate the incident light amplitude at a pixel level to form a Fresnel gray‐scale image. Similarly, it properly controls geometric phase modulation under the circularly polarized light (CP) incidence. For simplicity, we first consider the amplitude modulation, and for that, we designed an optical set‐up with a linear polarizer (set at 0°) and an orthogonal polarizer (set at 90°). When metasurface is placed in this orthogonal polarization path, the output light intensity “*I*” can be expressed as^[^ ^26^ ^]^

(1) $$I\;\left(\theta \right)=I_{o}\mathrm{si}n^{2}\;2\theta$$

where *I_o_* is incident light intensity and θ represents the orientation of each nanoresonator. (Section 1, Supporting Information). When θ varies from 0 to *π*/4, we get a continuous amplitude modulation varies from 0 to *I_o_* (depicted in **Figure** **3**h). Equation (1) dictates there are four–four possible orientation positions for the nanoresonator to produce maximum and minimum amplitude beam intensity. This one‐to‐many mapping relation of intensity and orientation provides an extra degree of freedom to manipulate the geometric phase of incident CP light. Resultantly, these orientation angles produce different geometric phases for phase manipulation. Furthermore, the efficient spin decoupling technique enables us to encode two independent information in this geometric phase modulation on a single metasurface.

Our geometric phase manipulation technique originates from the intrinsic characteristic of an anisotropic nanoresonator that provides two equal but opposite phase profiles for orthogonal incident CP light. This provides an extra degree of freedom to realize spin‐decoupling features. As a result, two nonidentical images can be encoded and displayed just by flipping the incident polarization handedness. If a CP light is an incident on an anisotropic nanoresonator from the forward direction, then the total geometric phase profile for both LHCP and RHCP components can be written as^[^ ^32^ ^]^:

(2) $$\varphi_{t}=\arg\left[\exp\left(i\varphi_{\mathrm{LHCP}}\right)+\exp\left(-i\varphi_{\mathrm{RHCP}}\right)\right]\;$$

where *φ*_*t*_, *φ*_LCP_, and *φ*_RCP_ are the total phase and phases profile for LHCP and RHCP incidence, respectively. By solving Equation (2) to get a complete phase profile for two distinct information encoding, (Section 2, Supporting Information) we get

(3) $$\varphi_{t}=\;\arg[\exp i.\left(\mathrm{ta}n^{-1}\left[\tan\left(\frac{\varphi_{\mathrm{LHCP}}-\varphi_{\mathrm{RHCP}}}{2}\right)\right)\right]$$

Equation (3) provides a completely merged phase profile (*φ*_*t*_) of two intended information required to encode on the metasurface interface. Contrary to the previously reported phase‐mergence technique‐based tri‐functional metasurfaces, our design technique is very simple and unique and requires only a geometric phase to encrypt the total phase profiles. These encoded phase profiles can be decrypted to project two holographic information just by shining the CP light. Projected holographic information will be flipped when incident CP light polarization is varied to orthogonally polarized light.

### 2.2 Optimization of Nanoresonator

Subwavelength size anisotropic meta‐nanoresonators have a strong localized effect on the phase and amplitude rendering of the incident wave. Their half‐waveplate nature provides great flexibility to design helicity‐dependent spin‐decoupling features. We employed an array of such identical nanoresonators made of a‐Si:H over the top of the SiO_2_ substrate. High‐extinction coefficient of *α*‐Si makes it unsuitable for visible band applications. We lowered its extinction coefficient by mixing the high hydrogen content, which eliminated the gap‐states in between the unstructured bonds by passivation. This hydrogen inclusion provides a constant bonding length, increases the mobility gap, and makes it transparent for the whole visible region. Measured complex refractive index of newly optimized a‐Si:H are 3.21 + 0.053i, 3.06 + 0.01i, and 2.88 + 0.001i at *λ* = 488 nm, *λ* = 532 nm, and *λ* = 633 nm.

To achieve maximum half‐waveplate functionality, the physical dimensions of the meta‐nanoresonator are optimized for minimum co‐polarized transmission and maximum cross‐polarized transmission efficiency at all operating wavelengths under the CP incidence. For optimization, first of all, the period (P) of the nanoresonator is optimized by rotating the nanoresonator for maximum cross‐polarized transmission, and the results are depicted in Figure S2 Supporting Information (Section 3,). By keeping in mind, the Nyquist sampling criteria *P* = 290 nm is selected as an optimal value. In the second step, other physical parameters, i.e., length (L) and width (w) of the nanoresonator, are optimized by keeping the period (*P* = 290 nm) and height (*H* = 400 nm) fixed. Obtained co‐ and cross‐polarized transmission efficiencies are depicted in Figure 3a–f. A white star is placed on the selected dimension of the nanoresonator with *w* = 90 nm, and *L* = 220 nm, which fulfills the minimum copolarized and maximum cross‐polarized transmission efficiency criteria. Complete 0–2*π* phase coverage is attained by rotating (0‐*π*) the selected nanoresonator about its own axis, enabling the geometric phase associated with anisotropic geometries. Low extinction‐coefficient and high material index support strong electromagnetic dipole resonances at all operating wavelengths. Resonances plots for all operational wavelengths are depicted in Figure S3–S5, Supporting Information (Section 4). These strong resonances give rise to high cross‐polarized transmission efficiency, as depicted in Figure 3g, and strongly validate our optimization procedure.

### 2.3 Fabrication and Results

**Figure 4:**
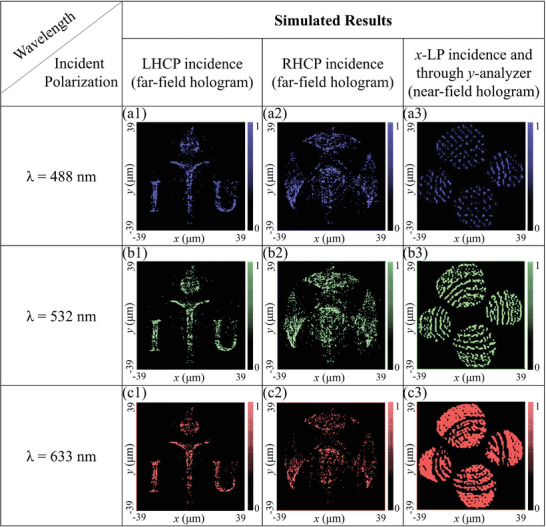
Simulated results of the tri‐functional metasurface. When LHCP wave is incident on the metasurface, it produces a holographic image of “ITU logo” in the far‐field, whereas it produces the “POSTECH logo” upon RHCP incidence. However, the “KAUST logo” can be observed through a *y*‐analyzer in the near field of metasurface upon *x*‐LP incidence. a1–a3) depicts results for *λ* = 488 nm, b1–b3) for *λ* = 532 nm, and c1–c3) for *λ* = 633 nm incidence.

To verify our proposed design strategy for trifunctional metasurface, we designed and simulated a 78.3 × 78.3 µm^2^ metasurface. First of all, two high‐resolution logos of “POSTECH” and “ITU” are selected, and their phase profile for 270 × 270 unit elements is numerically calculated in the far‐field through a modified Gerchberg–Saxton (GS) algorithm. A pictorial depiction of these calculated phase profiles is depicted in Figure S6, Supporting Information (Section 5). Then, these calculated phase maps are processed through Equation (3) in order to get a multiplexed phase profile, which can be implemented through a spin‐decoupling design strategy based on the geometric phase. Meanwhile, we calculated the amplitude profile of the gray‐scale “KAUST” logo for 78.3 × 78.3 µm^2^ metasurface with a pixel size of 290 × 290 nm^2^. This calculated amplitude profile is depicted in Figure S6, Supporting Information (Section 5). In the second step, this calculated amplitude profile is discretized by Equation (1) into four different orientation options (θ, π/2−θ, π/2+θ, and π−θ) for Malus's law degeneracy. While designing the metasurface, an exact orientation of the nanoresonator is finalized by rounding the calculated geometric phase profile to the closest available options (2θ, π−2θ, π+2θ, and 2π−2θ). Therefore, both intensity and phase profile can be modulated with one near‐field and two far‐field images on a single metasurface with a single meta‐nanoresonator (detailed design flow is depicted in Figure 2). This designed metasurface is simulated through a commercially available finite difference time domain (FDTD) solver. Simulated results for all operating wavelengths (*λ* = 488 nm, *λ* = 532 nm, and *λ* = 633 nm) are depicted in **Figure** **4**a1–c3. When LHCP is incident on the metasurface, it produces a far‐field holographic image of the “ITU logo” whereas a “POSTECH Logo” is depicted in the far‐field (focusing point 39.15 µm) on the RHCP incidence. A holographic image of the “KAUST logo” is depicted in the near field (sub‐wavelength scale) of the metasurface when analyzed through a *y‐*polarized analyzer, upon *x‐*LP is incidence.

**Figure 5:**
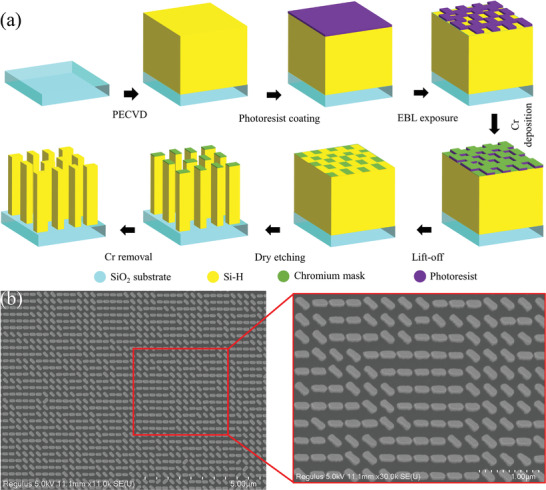
Fabrication steps of proposed tri‐functional metasurface and SEM images. a) All fabrication steps are explained. PECVD process is used for a‐Si:H deposition over the SiO_2_ substrate. Required patterns are transferred over the sample through EBL exposure on a thin layer of photoresist. The thin layer of chrome is deposited by electron beam evaporation. Lift‐off and dry etching processes are done to complete the fabrication process. b) SEM image of the fabricated sample of the tri‐functional metasurface. The inset shows a magnified view of the scale of 1000 nm.

We fabricated a sample of 500 × 500 µm2 with 1724 × 1724 unit elements matrix for experimental validation of tri‐functional metasurface using conventional electron beam lithography (EBL). Pictorial depiction of encoded phase plots and amplitude profile of encoded pieces of information is placed in Figure S7, Supporting Information (Section 5). For the fabrication process, first a film of 400 nm thickness of a‐Si:H is deposited on a SiO_2_ substrate of 2 × 2 cm^2^ size using a plasma‐enhanced chemical vapor deposition (PECVD) process. The main chamber's temperature (T) and pressure (p) remain fixed at 200 °C and 45 mTorr during the PECVD process. Next, a positive electron‐beam resistant (Microchem, PMMA 495 A6) is layered over the deposited a‐Si:H. The sample is rotated for 60 s at 2000 rpm^−1^. The sample is heated over a backing plate at 180 °C for 5 min. EBL (Elionix, ELS‐7800) is used for required pattern marking on the photoresist. The sample is immersed for 12 min in an isopropyl alcohol (CH_3_CHOHCH_3_)/methyl isobutyl ketone (C_6_H_12_O) solution at 0 °C to remove the EBL exposed photoresist. Next, a chromium (Cr) layer of 40 nm thickness is deposited with electron‐beam evaporation, and the sample is immersed for 12 h in hot acetone solution. Uncovered places of a‐Si:H are removed using plasma reactive ion etching. Finally, Cr etchant (CR‐7) is used to remove the Cr layer. Pictorial depiction of all fabrication steps and scanning‐electron microscope (SEM) images are depicted in **Figure** **5**.

**Figure 6:**
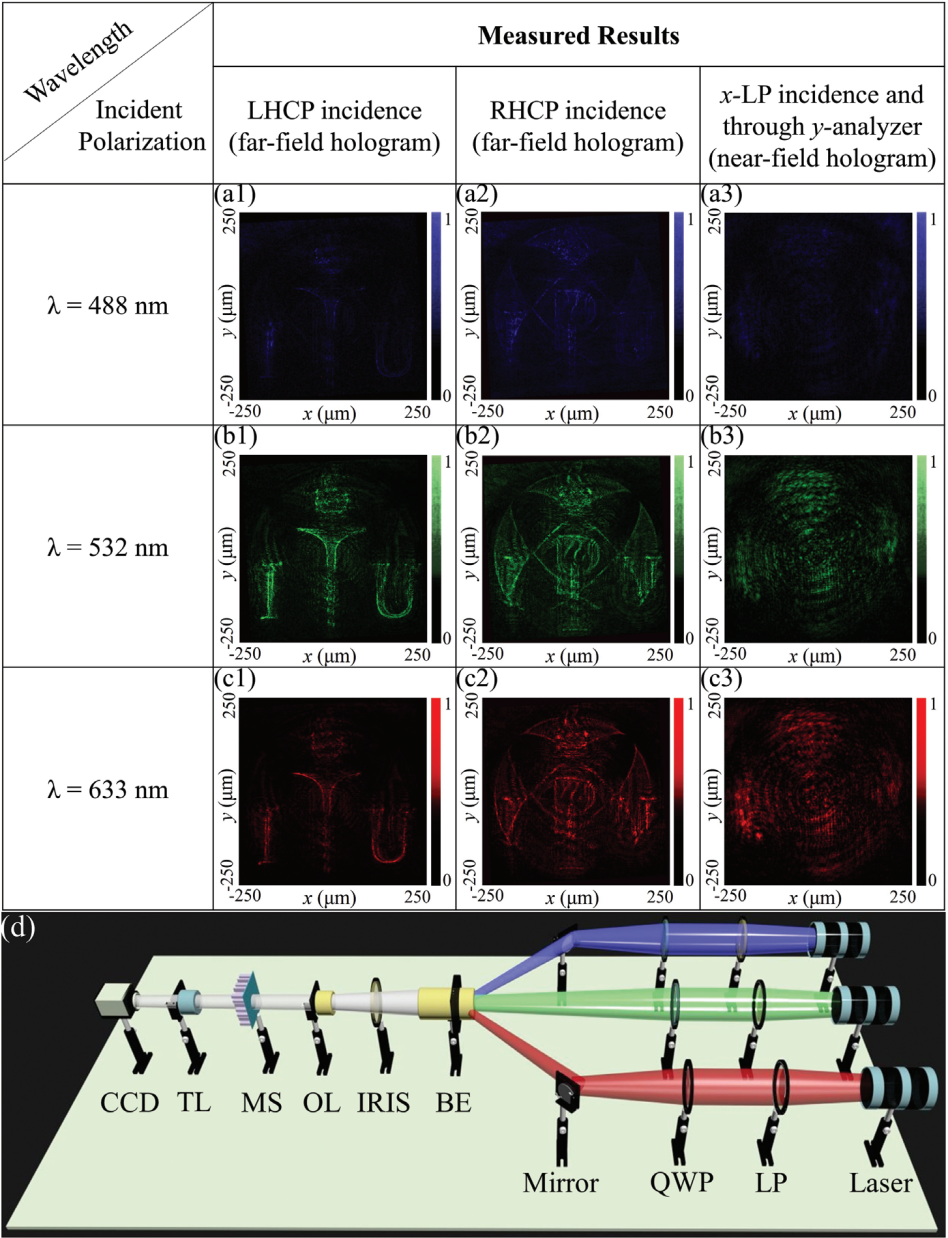
Far‐field and near‐field optically measured results of the proposed tri‐functional metasurface. When LHCP wave is incident on the metasurface, it produces a holographic image of “ITU logo” in the far‐field, whereas it produces the “POSTECH logo” upon RHCP incidence. However, the “KAUST logo” can be observed through a *y*‐analyzer in the near field of metasurface upon *x*‐LP incidence. a1–a3) depicts results for *λ* = 488 nm, b1–b3) for *λ* = 532 nm, and c1–c3) for *λ* = 633 nm incidence. d) Optical set‐up depiction, used for characterization the fabricated sample.

We used two different optical characterization set‐ups to test the near‐field and far‐field functionality of the proposed tri‐functional metasurface. Used optical set‐up for testing the metasurface working under CP light incidence is depicted in **Figure** **6**d. Three different laser sources of *λ* = 633 nm, *λ* = 532 nm, and *λ* = 488 nm are used for red, green, and blue colors. Incident laser beams are passed through a linear polarizer (LP) and a quarter‐waveplate polarizer (QP), and required polarization (LHCP or RHCP) is achieved by tuning the polarization axis of LP and the fast‐axis of QP. After setting the polarization axis, a beam is passed through the beam expander (BE), which collimates and expands the beam waist. Next, the beam is passed through the iris (I), which controls the waist of the beam. After setting an appropriate beam waist, it passed through an objective lens (OL) that focuses on the square shape metasurface. When incident beam polarization is set at LHCP proposed metasurface produces a holographic image of the “ITU logo” in the far‐field (focusing point 250 µm) of the metasurface. These far‐field outputs are focused through a tube lens (TL) on a charge‐coupled device (CCD) to record the images. The near‐field hologram is characterized by switching several optical components. The collimated laser beam is incident to a LP to produce the x‐LP light. The normally incident *x*‐LP beam transmits through a fabricated metasurface, then the near‐field transmission is collected by an OL. To choose the conjugate *y*‐LP beam, an analyzer is placed in between the OL and TL. Therefore, the CCD camera recorded only an analyzed component corresponding to near‐field output, “KAUST” logo. However, when incident polarization is tunned from LHCP to RHCP, the resultant far‐field holographic image is switched with the “POSTECH Logo” by validating the design theoretically predicted and simulated results. These holographic images have high resolution and image fidelity. These far‐field and near‐field measured results are depicted in Figure 6a1–c3. However, results depict some background noise in the measured results, and their one possible reason can be the discrete phase mapping to the amplitude modulation. The second reason is the imperfect intended polarization conversion of the incident unpolarized wave. This issue can be minimized by using more sophisticated optical elements.

## 3 Discussion

The proposed single meta‐nanoresonator‐based tri‐functional metasurface designing technique provides a number of technical advantages over previously reported designing techniques. We used a single nanoresonator (fixed height, width, length, and period) to encrypt three independent pieces of information on the metasurface interface by intelligently manipulating the phase and near‐field intensity modulation. However, the previously reported works have complex designing techniques leading to the complex fabrication process or unavoidable cross‐talk between shared multiple channels. A detailed comparative analysis with previously reported works is presented in Table S1, Supporting Information (Section 6). Therefore, the proposed design technique becomes an excellent candidate to avoid the aforementioned constraints while keeping the high information density for high‐dense optical storage.

Second, with the proposed‐designing method, three different optical set‐ups are required to decode three independent pieces of information, making it a potential candidate for anti‐counterfeiting applications. Specifically, far‐field holographic pieces of information need different helicities of incident CP light to project holographic information, whereas near‐field information can only be decoded through an orthogonally polarized light path. This provides an advantage to cipher secret messages in the near‐field (not observable by eye in the far‐field). This makes our proposed metasurface an excellent choice for making a multi‐fold anti‐counterfeiting strategy to increase the security of precious products.

Last, but not least, CMOS compatible platform of highly hydrogen‐enriched a‐Si:H with a broadband response for the entire visible regime brings promising practical applications. The proposed fabrication process provides almost comparable results to other dielectric platforms like gallium nitride (GaN) and titanium dioxide (TiO_2_) even without entangling with a high‐aspect‐ratio and complex fabrication issues. Moreover, transparent response for the entire visible regime and compatibility with the already matured semiconductor industry further adds numerous applications, including chip‐integrated displays and high‐density chip‐integrated optical storage.

## 4 Conclusion

In summary, we proposed and demonstrated a novel and simple design strategy for a tri‐functional metasurface by combining the geometric phase modulation and Malus's law amplitude modulation. Unlike the multi‐layer and super‐cell approaches, the proposed strategy employs a single meta‐nanoresonator to encode multiple information on the metasurface interface. Due to the combination of multiple optical properties manipulation, the proposed design strategy provides great freedom to independently manipulate each channel for information encoding. Resultantly, the proposed sample projects two far‐field meta‐holography images and one near‐field nano‐printing image. The advantages of cross‐talk free channels, simple design & fabrication technique, high‐resolution outputs, and ultra‐compact size make the proposed meta‐device an excellent choice for many high‐end applications like high‐dense information encoding, multi‐channel displays, AR/VR, and anti‐counterfeiting, etc.

## Conflict of Interest

The authors declare no conflict of interest.

## Author Contributions

M.Q.M, J.S., M.A.N., and J.K. contributed equally to this work. J.R., M.Q.M., and M.A.N. conceived the idea and initiated the project. M.A.N., and M.Q.M. designed the holograms and performed the numerical simulations. J.S. and J.K. designed the process, experiments and fabricated the device. M.Z. analyzed the data. M.A.N. and M.Q.M. mainly wrote the manuscript. J.R. and Y.M. guided the entire research. All authors participated in the discussion and confirmed the final manuscript.

## Supporting information

Supporting Information

## Data Availability

The data that support the findings of this study are available from the corresponding author upon reasonable request.
