# Supplementary material for: Single‐Cell‐Driven Tri‐Channel Encryption Meta‐Displays
Source: Adv Sci (Weinh). 2022 Oct 26;9(35):2203962. doi: 10.1002/advs.202203962 (PMC9762282; doi:10.1002/advs.202203962)
Supplement: Supplementary file 1 — Supporting Information [file ADVS-9-2203962-s001.pdf]

ADVANCED SCIENCE
Open Access

Supporting Information

for *Adv. Sci.*, DOI 10.1002/advs.202203962

Single-Cell-Driven Tri-Channel Encryption Meta-Displays

Muhammad Qasim Mehmood, Junhwa Seong, Muhammad Ashar Naveed, Joohoon Kim, Muhammad Zubair, Kashif Riaz*, Yehia Massoud* and Junsuk Rho**

Supporting Information

Single-Cell-Driven Tri-Channel Encryption Meta-Displays

*Muhammad Qasim Mehmood[‡,a], Junhwa Seong[‡,b], Muhammad Ashar Naveed[‡,a], Joohoon Kim[‡,b], Muhammad Zubair[*a], Kashif Riaz[*a], Yehia Massoud[*,c], Junsuk Rho[*bdef]*

Muhammad Qasim Mehmood[‡], Muhammad Ashar Naveed[‡], Muhammad Zubair, Kashif Riaz
[a]MicroNano Lab, Electrical Engineering Department, Information Technology University (ITU) of the Punjab, Ferozepur Road, Lahore 54600, Pakistan
E-mail: kashif.riaz@itu.edu.pk, muhammad.zubair@itu.edu.pk

Junhwa Seong[‡], Joohoon Kim[‡], Prof. Junsuk Rho
[b]Department of Mechanical Engineering, Pohang University of Science and Technology (POSTECH), Pohang 37673, Republic of Korea
E-mail: jsrho@postech.ac.kr

Yehia Massoud
[c]Innovative Technologies Laboratories (ITL), King Abdullah University of Science and Technology (KAUST), Thuwal 23955, Saudi Arabia.
E-mail: yehia.massoud@kaust.edu.sa

Prof. Junsuk Rho
[d]Department of Chemical Engineering, Pohang University of Science and Technology (POSTECH), Pohang 37673, Republic of Korea

Prof. Junsuk Rho
[e]POSCO-POSTECH-RIST Convergence Research Center for Flat Optics and Metaphotonics, Pohang 37673, Republic of Korea

Prof. Junsuk Rho
[f]National Institute of Nanomaterials Technology (NINT), Pohang 37673, Republic of Korea

‡ These authors have contributed equally to this work.

Keywords: Tri-functional Metasurface, Metahologram, High-dense Meta-optics, Phase-amplitude Modulation, Meta-displays.

Section 1: Principle of amplitude-modulation based gray-scale image

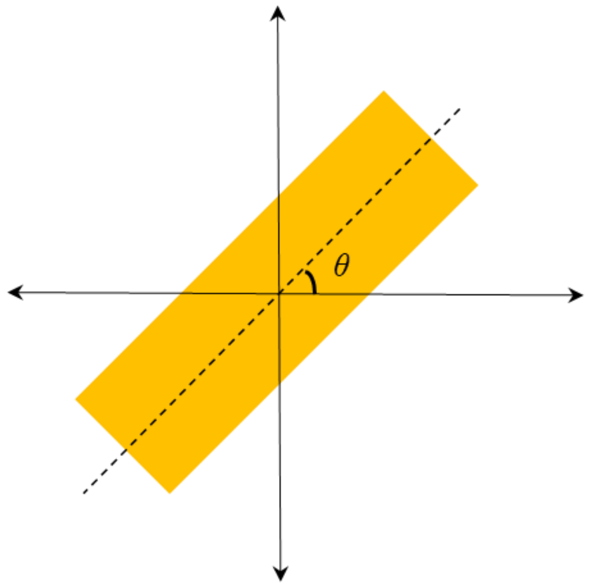

Figure S1: An-isotropic meta-nanoresonator rotated at an angle "θ" w.r.t. *x*-axis.

The Jones matrix of a meta-nanoresonator with an in-plane orientation angle θ can be expressed as

$$\boldsymbol{J(\theta) = R(-\theta)J_0R(\theta)} = \begin{bmatrix} \mathbf{cos\theta} & \mathbf{-sin\theta} \\ \mathbf{sin\theta} & \mathbf{cos\theta} \end{bmatrix} \begin{bmatrix} \boldsymbol{t_l} & \mathbf{0} \\ \mathbf{0} & \boldsymbol{t_s} \end{bmatrix} \begin{bmatrix} \mathbf{cos\theta} & \mathbf{sin\theta} \\ \mathbf{-sin\theta} & \mathbf{cos\theta} \end{bmatrix} \tag{ES1}$$

where *R(θ)* is the rotation matrix, t_l and t_s are complex transmission coefficients when an incident light beam is polarized along the long and short axes of the nanoresonator, respectively. If an incident light with LXP polarized amplitude is incident on this nanoresontor and we record/visulize it's response through an analyzer which is polarized in LYP direction, then transmission jones matrix will be transform as:

$$\boldsymbol{T_n(\theta)} = \begin{bmatrix} \mathbf{0} & \mathbf{0} \\ \mathbf{0} & \mathbf{1} \end{bmatrix} \boldsymbol{J(\theta)} \begin{bmatrix} \mathbf{1} \\ \mathbf{0} \end{bmatrix} = \frac{\boldsymbol{t_l - t_s}}{\mathbf{2}} \begin{bmatrix} \mathbf{0} \\ \boldsymbol{sin2\theta} \end{bmatrix} \tag{ES2}$$

Thus, the transmitted light intensity can be expressed as

$$\boldsymbol{I(\theta) = T_{cross}Sin^2 2\theta} \tag{ES3}$$

where $T_{cross} = \left|\frac{t_l - t_s}{2}\right|^2$.

If we solve this equation for maximum intensity, and minimum intensity we will get a continuosly varrying angle θ. Which gives minimum value at 0, 90, 180, and 360 degree. On the otherhand it provides maximum value at 45, 135, 225 and 315.

Section 2: Spin-decoupling strategy for two-independent holographic images encryption

Suppose we have two phase profiles, of two images, and we want to implement one phase for left circularly polarized (φ_{LHCP}) and second for right circularly polarized light (φ_{RHCP}), keeping the incident direction of light fix. So total phase profile need to be implemented on whole metasurface will be

$$\boldsymbol{\varphi_t = \arg[\exp(i\varphi_{LHCP}) + \exp(-i\varphi_{RHCP})]}. \quad \text{(ES4)}$$

By expanding this

$$\boldsymbol{\varphi_t = \arg[\mathrm{Cos}(\varphi_{LHCP}) + i\,\mathrm{Sin}(\varphi_{LHCP}) + \mathrm{Cos}(\varphi_{RHCP}) - i\,\mathrm{Sin}(\varphi_{RHCP})]} \quad \text{(ES5)}$$

$$\boldsymbol{\varphi_t = \arg[\mathrm{Cos}(\varphi_{LHCP}) + \mathrm{Cos}(\varphi_{RHCP}) + i\{\mathrm{Sin}(\varphi_{LHCP}) - \mathrm{Sin}(\varphi_{RHCP})\}]} \quad \text{(ES6)}$$

By solving this we can find a final expression of total phase which is needed to implement on metasurface.

$$\boldsymbol{\varphi_t = tan^{-1}[\frac{Sin(\varphi_{LHCP}) - Sin(\varphi_{RHCP})}{Cos(\varphi_{LHCP}) + Cos(\varphi_{RHCP})}]} \quad \text{(ES7)}$$

$$\varphi_t = tan^{-1}[\frac{Sin\left(\frac{\varphi_{LHCP} - \varphi_{RHCP}}{2}\right)}{Cos\left(\frac{\varphi_{LHCP} - \varphi_{RHCP}}{2}\right)}] \quad \text{(ES8)}$$

$$\varphi_t = tan^{-1}[tan\left(\frac{\varphi_{LHCP} - \varphi_{RHCP}}{2}\right)] \quad \text{(ES9)}$$

$$[\varphi_t = \arg[\exp i\,.\,(tan^{-1}[tan\left(\frac{\varphi_{LHCP} - \varphi_{RHCP}}{2}\right))] \quad \text{(ES10)}$$

Equation ES10, defines the total phase profile which we need to implement on the metasurface

Section 3: Period optimization of meta-nanoresonator

Period of proposed meta-nanoresonator is optimized by rotating it about its own-axis and period is contnueously varied for maximum cross-polarized transmission. Optimization results are depicted in Figure S2. P=290 nm is selected as optimized dimension. A black dashed line marks the selected dimension.

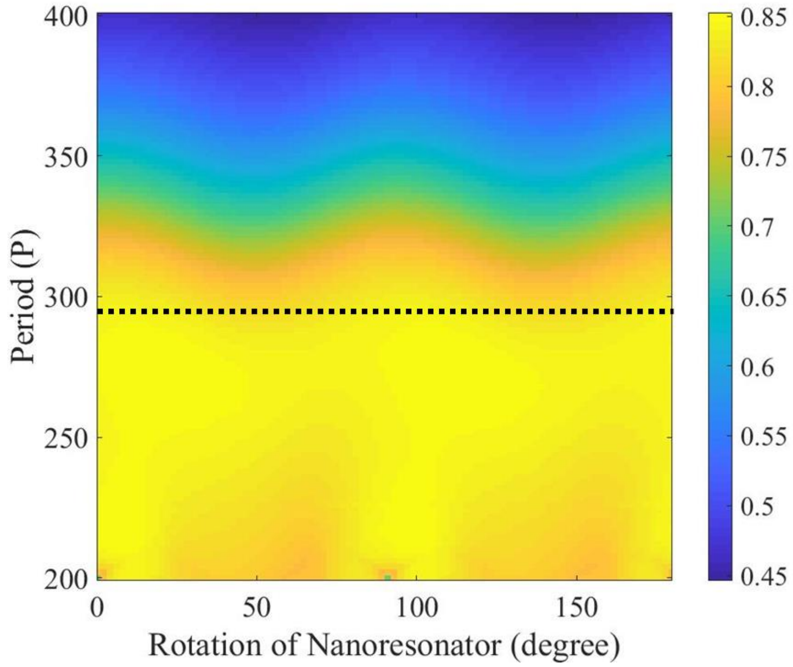

Figure S1: Efficiency of cross-polarization transmission for a range of P and rotation angles (θ) of the a-Si:H unit cell. The black dashed line at P = 290 nm denotes the selected dimension.

Section 4: Multiple resonance modes inside optimized meta-nanoresonator

Dielectric resonance modes for selected meta-nanoresonators at all operational wavelengths (λ=488 nm, λ=532 nm, and λ=633 nm) are depicted in Figures S3, S4, and S5. Both electric and magnetic resonances are well confined inside the meta-nanoresonator.

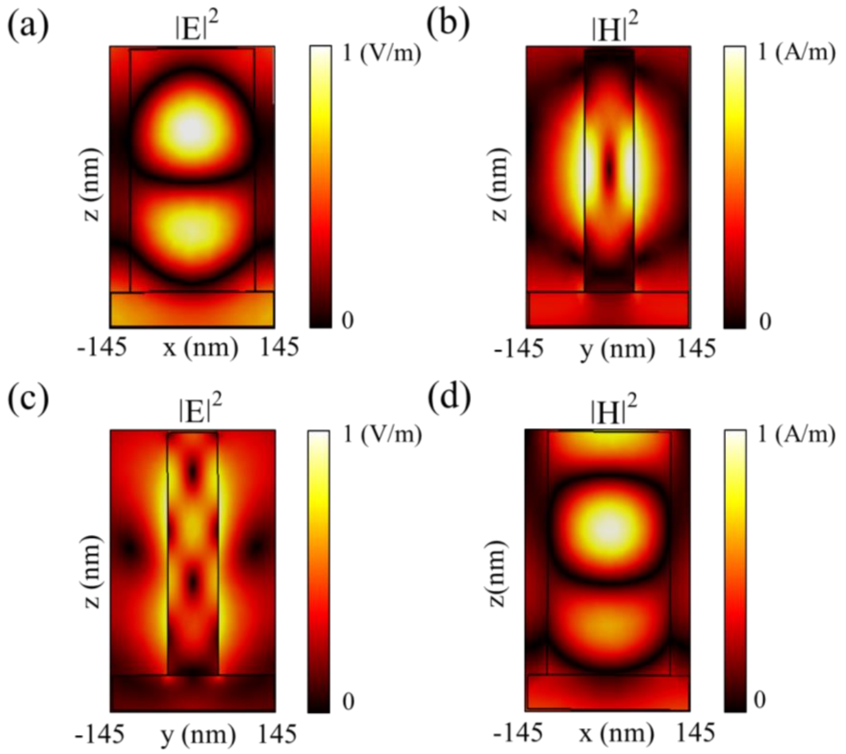

Figure S3: Resonance modes inside the a-Si:H meta-nanoresonators at λ = 488 nm. Cross-sectional view of field intensities and orientations under *x*-polarized incident light for the (a) electric field and (b) magnetic field. Cross-sectional view of field intensities and orientations under *y*-polarized incident light for the (c) electric field and (d) magnetic field.

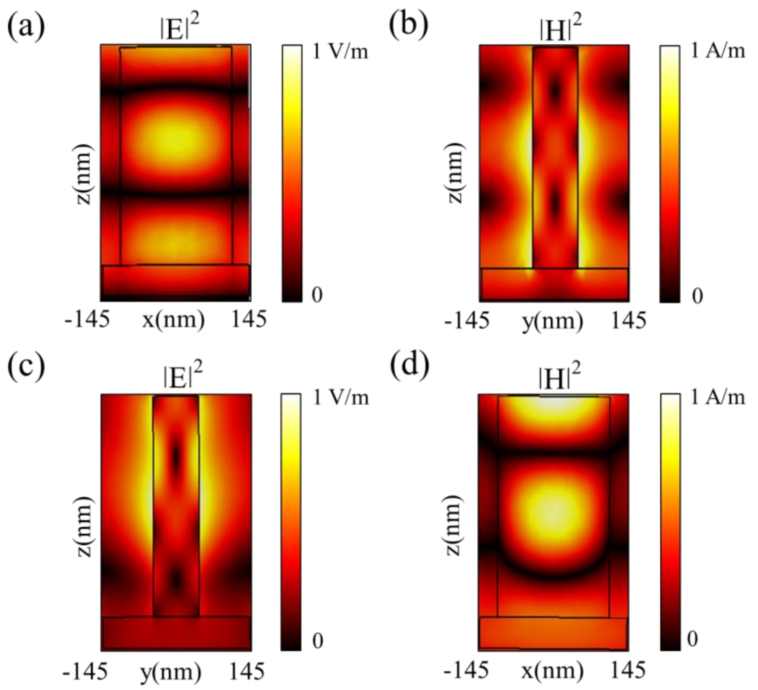

Figure S4: Resonance modes inside a-Si:H meta-nanoresonators *at* λ = 532 nm. Cross-sectional view of field intensities and orientations under x-polarized incident light (a) Electric field (Vm^{-1}) (b) Magnetic field (Am^{-1}). Cross-sectional view of field intensities and orientations under y-polarized incident light (c) Electric field (Vm^{-1}) (d) Magnetic field (Am^{-1}).

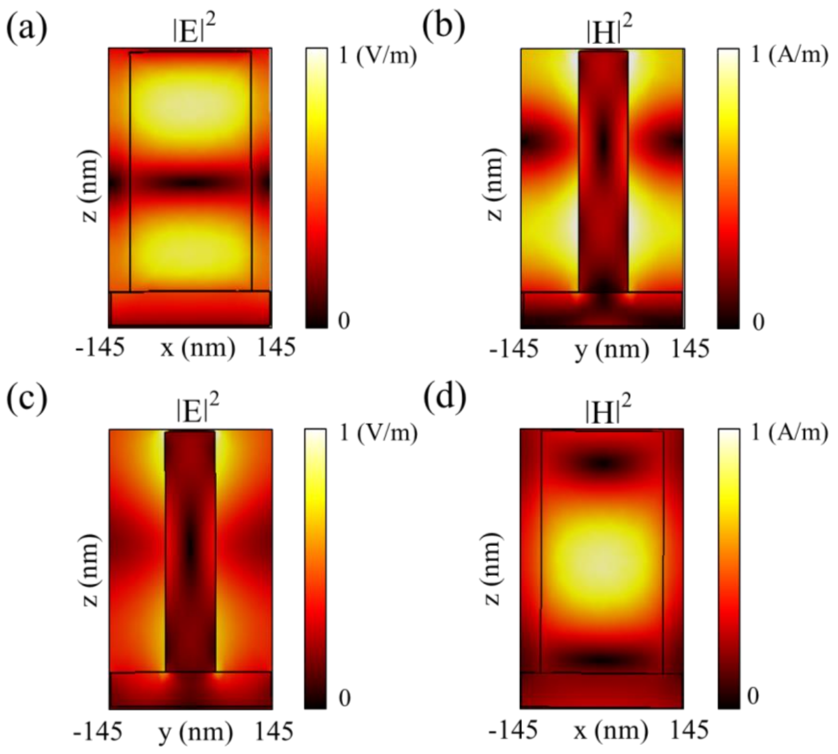

Figure S5: Resonance modes inside a-Si:H meta-nanoresonators *at* λ = 633 nm. Cross-sectional view of field intensities and orientations under x-polarized incident light (a) Electric field (Vm^{-1}) (b) Magnetic field (Am^{-1}). Cross-sectional view of field intensities and

orientations under y-polarized incident light (c) Electric field (Vm^{-1}) (d) Magnetic field (Am^{-1}).

Section 5: Numerically caculated results

Numerically calculated phase plots far-field metaholograms for two images ("POSTECH logo" and "ITU Logo") under right and left circularly polarized light and near-field amplitude plot of "KAUST logo" are depicted below in Figure S6. A modified Gerchberg-Saxton (GS) algorithm is used to calculate these phases for a 78.3 × 78.3 μm^2 metasurface.

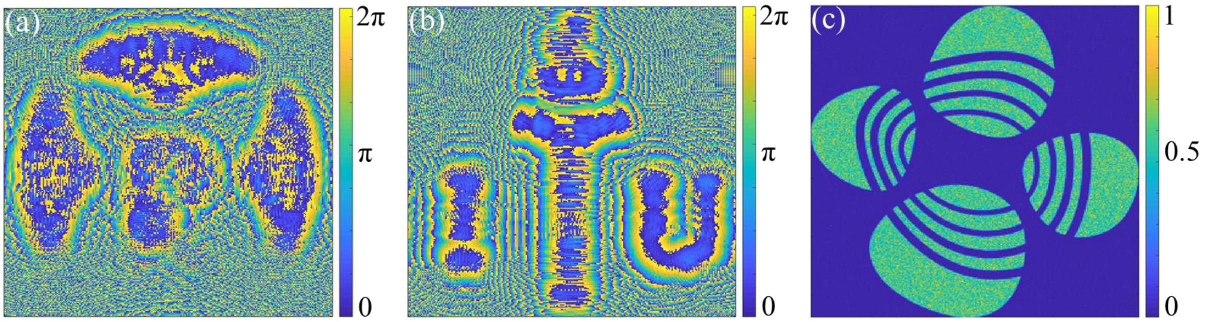

Figure S6: Numerically calculated phase and amplitude of for different logos for 270×270 array of meta-nanoresonators (a) Phase-map of POSTECH logo calculated for RHCP, (b) phase-map of ITU logo calculated for LHCP. (c) depicts amplitude modulation for KAUST logo.

Numerically calculated phase plots far-field metaholograms for two images ("POSTECH logo" and "ITU Logo") under right and left circularly polarized light and near-field amplitude plot of "KAUST logo" are depicted below in Figure S7. A modified Gerchberg-Saxton (GS) algorithm is used to calculate these phases for a ≈ 500 × 500 μm^2 metasurface.

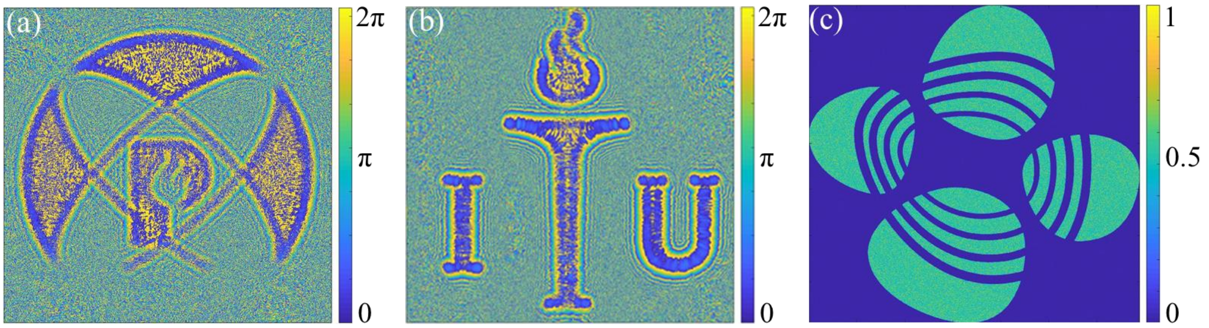

Figure S7: Numerically calculated phase and amplitude of for different logos for 1724×1724 array of meta-nanoresonators (a) Phase-map of POSTECH logo calculated for RHCP, (b) phase-map of ITU logo calculated for LHCP. (c) depicts amplitude modulation for KAUST logo.

Section 6: Technology review

A detailed technology review is presented in Table S1 to make a comparative analysis of this work with previously reported works.

Table S1. Comparative analysis of reported tri-functional metasurface with the present study.

**Decoupling Level**	**No of Channels**	**Platform**	**Main Approach**	**Features**	**Design Complexity**
No Decoupling [1]	3	Plasmonic (Al)	Stacking	Inevitable cross-talk and low efficiency	High
No Decoupling [2]	2	All-dielectric (Si/SiO_2)	Segmenting	Inevitable cross-talk and low efficiency	Partially high
No Decoupling [3]	3	Plasmonic (Al)	Interleaving	Inevitable cross-talk and low efficiency	Partially high
Partial decoupling [4]	2	Plasmonic (Ag)	Orientation degeneracy	Finite and unequidistant phase steps (2 or 4)	Low
Partial decoupling [5]	2	All-dielectric (Si/SiO_2)	Combining structural color with phase manipulation	Unwanted spectral amplitude or limited structural color	Partially high
Partial decoupling [6]	2	All-dielectric (Si/SiO_2)	Complex amplitude modulation	Interrelation between far-field phase-only functionality and amplitude control	Partially high
Complete decoupling [7]	2	All-dielectric (TiO_2/SiO_2)	Combining propagation phase, PB phase, and Malus's Law	Completely decoupling the near- and far-field functionality without sacrifice of efficiency	Partially high
Complete decoupling [8]	3	All-dielectric (TiO_2/SiO_2)	Combining propagation phase, PB phase, and Malus's Law	Completely decoupling the near- and far-field functionality without sacrifice of efficiency	Partially high
Complete decoupling [This Work]	3	All-dielectric (a-Si:H/SiO_2)	PB phase, and Malus's Law	Completely decoupling the near- and far-field functionality without sacrifice of efficiency	Low

Supplemental References

[1] X. Luo, Y. Hu, X. Li, Y. Jiang, Y. Wang, P. Dai, Q. Liu, Z. Shu, H. Duan, *Adv. Opt. Mater*. **2020**, 8, 1902020.

[2] R. Chen, Y. Zhou, W. Chen, R. Chen, N. Iqbal, Y. Ma, *ACS Photonics* **2020**, 7, 1171.

[3] C. Zhang, F. Dong, Y. Intaravanne, X. Zang, L. Xu, Z. Song, G. Zheng, W. Wang, W. Chu, X. Chen, *Phys. Rev. Appl*. **2019**, 12, 34028.

[4] L. Deng, J. Deng, Z. Guan, J. Tao, Y. Chen, Y. Yang, D. Zhang, J. Tang, Z. Li, Z. Li, S. Yu, G. Zheng, H. Xu, C.-W. Qiu, S. Zhang, *Light-Sci. Appl*. **2020**, 9, 101.

[5] Q. Wei, B. Sain, Y. Wang, B. Reineke, X. Li, L. Huang, T. Zentgraf, *Nano Lett*. **2019**, 19, 8964.

[6] A. C. Overvig, S. Shrestha, S. C. Malek, M. Lu, A. Stein, C, Z., N. Yu, *Light-Sci. Appl*. **2019**, 8, 92.

[7] J. Li, Y. Wang, C. Chen, R. Fu, Z. Zhou, Z. Li, G. Zheng, S. Yu, C.-W. Qiu, S. Zhang, *Adv. Mater*. **2021**, 33, 2007507.

[8] Z. Li, C. Chen, Z. Guan, J. Tao, S. Chang, Q. Dai, Y. Xiao, Y. Cui, Y. Wang, S. Yu, G. Zheng, S. Zhang, *Laser Photonics Rev*. **2020**, 14, 2000032.
